# Medicine
^1^For the following abstracts and references I am indebted to Schmidt's Jahrbücher, 1902, cclxxiii. Heft. i.


**Published:** 1902-12

**Authors:** J. Michell Clarke


### Progress of the Medical Sciences.

### MEDICINE.[1]

**Fat-splitting Ferment in Stomach.** Of much interest is the discovery by Volhard[2] that the stomach contains a fat-splitting ferment, which, like pepsin and the rennet-ferment, is secreted by the fundus of the stomach. This ferment does not appear if unemulsified fats or oils are mixed with the watery contents of the stomach, but acts very powerfully on natural (yolk of egg, cream, milk) and artificial emulsions. Experiments show that the fat-splitting ferment corresponds entirely to pepsin and rennet-ferment, in its relations to acids, alkalies, &c., and follows the same changes in diseases of the stomach.

**Influence of Foods on Gastric Secretion.** The researches of Herzen, Radzikowsky and Mark-Schnorf, at Lausaune,[3] confirm the well-known conclusions of Pawlow. These researches show that some of the most generally used foods cause a flow of gastric-juice; *i.e.*, by a specific chemical action on the mucous membrane they so stimulate it that it at once begins to pour out an abundant secretion. These foods include red meats, meat-juice, broths, Liebig's extract, milk, gelatine, certain peptones, and water. The last acts only in large amount. It does not follow, however, as might be thought from the researches of Schiff, that secretion and pepsin formation always go together, are one and the same thing. This view is untenable; the substances which cause secretion of gastric-juice act through the nervous system, and have no action when introduced per rectum, whilst those which cause pepsin-formation act through the blood, and are efficient when absorbed per rectum. Herzen and his pupils have now shown that Liebig's extract acts more especially in causing secretion of juice, dextrin in formation of pepsin; *e.g.*, that these are food stuffs, which act in one only of these ways. Alcohol acts powerfully in the former, and vitellin and liver-glycogen in the latter way. These observations lead to many practical results; the physician may, according to the nature of the case, influence the quantity of gastric fluid secreted or the amount of pepsin formed. Favourable results have followed from the administration of a mixture of Liebig's extract and dextrin in suitable amounts.

[1] For the following abstracts and references I am indebted to Schmidt's *Jahrbücher*, 1902, cclxxiii. Heft. i.

[2] *Ztschr. f. klin. Med.*, 1901, xlii. 414; xliii. 397.

[3] *Arch. f. d. ges. Physiol.*, 1901, lxxxiv. 101, 513; lxxxv. 143 respectively.

**Ulcer of Stomach.** v. Yzeren[1] has proved by his experiments on puppies, that section of the vagi is followed after some days by the formation of an ulcer, which conforms in every respect to the ordinary stomach-ulcer in man. He concludes that spasmodic contraction of the pyloric region of the stomach is the cause of ulcer, and that it can only be cured with certainty by removing this spasm and its causes. Instead therefore of the dangerous and, in results, uncertain operation of gastro-enterostomy, he proposes a thorough division of the pyloric muscle-fibres down to the submucosa, a comparatively simple operation, which gave good results in his puppies.

**Blood-examination as a means of diagnosis between ulcer and cancer of the stomach.** Rencki[2] concludes that this is of no great practical use. In the case of the red corpuscles the fall of the hæmoglobin-content below 60 per cent. is not in favour of cancer, nor does the presence of nucleated red corpuscles negative ulcer; and with reference to the leucocytosis of digestion, one can only conclude from its non-appearance that there is some stomach disturbance, the one most generally associated with it being a relaxation or insufficiency of the pylorus.

**Hour-glass Stomach.** Büdinger[3] remarks that in this affection the passage of the chyme through the narrowed portion can often be plainly felt, and advises operation in such cases, the proper procedure in many cases being in his opinion the resection of the pyloric segment. He reports the following case in which an hour-glass condition of the stomach was due to muscular contraction:—

In a patient, æt. 42, in whom all the symptoms of hour-glass contraction of the stomach were present, the abdomen was opened, but only slight adhesions of the organ and a scar on the anterior wall were found. As this scar was being handled, a contraction began at the greater curvature with a strong drawing in of the lower border of the stomach, which, quickly and steadily increasing, advanced towards the pylorus, and was so strongly marked in the neighbourhood of the scar, somewhere about the sphincter antri pylori, as to produce a typical hour-glass deformity of the stomach. The contraction lasted about five seconds, and then passed off; the pylorus remaining quiet. This case shows that in addition to the contracture of the proper muscle at the pylorus in ulcer, hyper-acidity, &c., and to spasm of the whole pyloric region, which may be present constantly or intermittently and cause difficulty in diagnosis by resembling a tumour, we must also distinguish a special spasm of the sphincter antri pylori.

In the cases above referred to, in which there may be difficulty in distinguishing between a growth in the pylorus and

[1] *Ztschr. f. klin. Med.*, 1901, xliii. 181.
[2] *Arch. f. Verdauungskrankh.*, 1901, 234, 392.
[3] *Wien. klin. Wchnschr.*, 1901, xiv. 36.

the false tumour formed, by persistent spasm of the pyloric muscle, Bouveret[1] lays stress on the fact that in spasm the supposed tumour may be felt to disappear under the palpating finger.

As to the allied condition in sucklings, the so-called congenital pyloric stenosis of sucklings, there is much difference of opinion, some authors attributing the condition to spasm of the pyloric muscle, others to a true hypertrophy of the pylorus, while others again hold that both of these conditions are concerned.

**Cancer of the Stomach.** With regard to the question of the growth of cancer of the stomach from an old ulcer, Borrmann, in his recent work,[2] out of 63 cases examined *post-mortem* did not find one in which this mode of origin could certainly be attributed to the growth. Krokiewicz[3] also, in reporting a case of this kind, says that out of 7,000 *post-mortem* examinations in Lemberg and Cracow he had not seen one instance of carcinoma originating in ulcer. On the other hand, Boekelman[4] states that in the Netherlands the transition from ulcer to cancer is so frequent, that almost half the cases of cancer of the stomach originate in this way. He thinks that the conditions are altogether different from those in cases of primary cancer of the stomach. In favour of this is the difference in the state of the whole gastric mucous membrane; in primary cancer there are extensive interstitial changes, with marked glandular atrophy; in carcinoma starting from an ulcer these changes are very much less, and hence the corresponding retention of the secretory activity.

With regard to the bacilli found in the stomach-contents in cancer, Ehret[5] thinks that the presence of very numerous thread-like bacilli, in the absence of any especially great stagnation of the stomach contents, points with great probability to the diagnosis of cancer. Schmidt,[6] discussing the bacilli, whose presence in cancer of the stomach has been so frequently observed, has found that their development is favoured by stagnation of the stomach contents, by decrease in formation of HCl and ferments, and especially through mingling of albuminous detritus and blood with the contents of the stomach. This accounts for their frequency and abundance in cases of cancer. The presence of pseudo-lactic acid bacilli may lead to mistakes. It is remarkable that luxuriant growths of the bacillus coli may be found in carcinomatous stomachs, without there being any fistulous communication between the stomach and intestine. Boas[7] draws attention to the fact that careful investigation shows that blood is much more frequently found in

[1] *Lyon méd.*, 1901, Juin 23.
[2] *Das Wachsthum u. d. Verbreitungswege des Magencarcinoms.* Jena, 1901.
[3] *Wein. klin. Wchnschr.*, 1901, xiv. 8. [4] *Ztschr. f. klin. Med.*, 1902, xliv. 128.
[5] *Semaine méd.*, 1901, Mars 6. [6] *Wien. klin. Wchnschr.*, 1901, xiv. 2.
[7] *Deutche. med. Wchnschr.*, 1901, xxvii. 20.

the contents of the stomach in cancer than is generally recognised, though very often in small amounts only recognisable microscopically. He thinks that these "occult hemorrhages" are not only of considerable value in diagnosis, since outside cancer they are only found in ulcer and in pyloric stenosis, but that they also have an important influence on the course of the disease, the bad effect of constant small hemorrhages being well known.

**Gastrosuccorhœa. Reichmann's Disease.** v. Aldor[1] contends for the recognition of this malady as a disease *sui generis*. It must be clearly delimited. It has nothing in common with cases in which small quantities of fluid, often containing HCl, but never pepsin, may be found in healthy stomachs, nor with those in which there is an excessive secretion of gastric-juice in consequence of motor disturbances, nor with the periodic hypersecretion in gastroxynsis; *e.g.*, in gastric crises of tabes. There remain the not very frequent but well-defined cases in which the symptoms of severe gastric pain, frequent vomiting of large amounts, great thirst, persistent constipation, form a very obstinate malady whose nature can only be correctly diagnosed by careful and repeated examination of the stomach contents both during fasting and after meals. Treatment consists in the administration of hot alkaline saline waters, washing out of the stomach, and a careful dietary with abundant fat. Atropin is beneficial, morphia harmful.

J. MICHELL CLARKE.

[1] *Berl. klin. Wchnschr.*, 1901, xxxviii. 39.

